# Acid-sensing ion channels mediate the degeneration of intervertebral disc via various pathways—A systematic review

**DOI:** 10.1080/19336950.2019.1664038

**Published:** 2019-09-17

**Authors:** Yingjun Guo, Yang Meng, Hao Liu, Beiyu Wang, Chen Ding, Xin Rong, Yi Yang, Ying Hong

**Affiliations:** Department of Orthopedic Surgery, West China Hospital, Sichuan University, Chengdu, Sichuan Province, China

**Keywords:** Acid-sensing ion channel, intervertebral disc degeneration, hypoxia, apoptosis

## Abstract

To elucidate the pathological significance of acid-sensing ion channels (ASICs) in intervertebral disc degeneration (IVDD), the database of Medline, Web of Science, and EmBase were carefully screened. Search terms used in each database varied slightly to optimize results. Data relating to the correlation between ASICs and IVDD was systematically collected and integrated into the review. 11 basic science studies, containing the related information, were finally identified for inclusion. Intervertebral disc degeneration (IVDD) is a common disease in middle-aged and elderly people, which has a great impact on patients’ quality of life. Many research teams have attempted to elucidate the pathogenesis of this degenerative disease, and have made considerable progress. Acid-sensing ion channels (ASICs) were once reported to be able to regulate the apoptosis process of chondrocytes in joint cartilage, which has been transplanted into the IVDD-related research. ASIC1a functions as the mediator for cells in nucleus pulposus (NP) and endplate (EP), with whose activation the apoptosis process would be accelerated. Moreover, ASIC1a’s activation could also regulate the anabolism in chondrocytes of EP, facilitating the degeneration. ASIC3 would only promote the degeneration in NP, possibly via its pro-inflammatory effect. The distribution of ASICs in NP, EP, annulus fibrosus, and the particular functions of ASIC1a and ASIC3 remind us about the pathological significance of ASICs in IVDD, which could be a promising therapeutic target in future treatment for IVDD.

## Introduction

Intervertebral disc degeneration (IVDD) is a major cause of cervical spondylosis and low back pain, and despite its long history and large socio-economic burden, the initiation and progress of disc degeneration is not well understood^[1]^. The intervertebral disc (IVD) is a viscoelastic weight-bearing “cushion” and plays a crucial role in the maintaining flexibility and stability of the spine, which is localized between adjacent spinal vertebrae [2], consisting of three morphologically distinct regions: endplates (EP), annulus fibrosus (AF), and nucleus pulposus (NP) [3]. EP has been reported to be able to absorb nutrients and deliver them to other disc parts. The AF is circular exterior, surrounding the soft NP, which may prevent the NP from herniating of the disc by hydraulically sealing the nucleus and by distributing the pressure and force imposed on the IVD [4,5]. As they age, the degenerative EP causes the malnutrition of NP and the dysfunction of NP cell (NPC) gradually, leading to the decrease of the disc’s elasticity and the instability, which represent the well-accepted etiology of IVDD.

Acid-sensitive ion currents were firstly reported in sensory neurons, which could be triggered by the decrease of pH, and it has been known that the acid-sensing ion channels (ASICs), belonging to the degenerin/epithelial Na+ channel family, were responsible for this current [6]. Currently, six subunits have been identified: ASIC1a, ASIC1b, ASIC2a, ASIC2b, ASIC3, and ASIC4, forming the homomeric or heteromeric channel complexes [7]. ASICs expressed widely in various tissues in the body and its activation plays an important regulatory role in many physiological or pathological processes.

Physiologically, the disc is located in a specific microenvironment of hypoxia and acidity, and the NP represents the largest hypoxic tissue in human body [8]. This special microenvironment is of significance for NPC growth and metabolism, and the acidity plays a central role in inhibiting the cellular activity and physiological function, while it is aggravated during the IVDD [9,10]. Recently, increasing studies proved the activation of ASICs in IVD, and in this systematic review, we try to explain the correlation between ASICs and IVDD.

## Methods

### Search strategy

A computerized search was performed in Medline, Web of Science, and EmBase from the inception of each database to December 15^th^, 2018, using the following searching keywords:

(((((intervertebral disc[Title/Abstract]) OR nucleus pulposus[Title/Abstract]) OR annulus fibrosus[Title/Abstract]) OR endplate[Title/Abstract])) AND ((((((((((((((((((((acid-sensing ion channels[Title/Abstract]) OR acid-sensing ion channel[Title/Abstract]) OR acid-sensing ion channel 1a[Title/Abstract]) OR acid-sensing ion channel 1b[Title/Abstract]) OR acid-sensing ion channel 2a[Title/Abstract]) OR acid-sensing ion channel 1b[Title/Abstract]) OR acid-sensing ion channel 3[Title/Abstract]) OR acid-sensing ion channel 4[Title/Abstract]) OR acid-sensing ion channel 1[Title/Abstract]) OR acid-sensing ion channel 2[Title/Abstract]) OR ASICs[Title/Abstract]) OR ASIC[Title/Abstract]) OR ASIC1a[Title/Abstract]) OR ASIC1b[Title/Abstract]) OR ASIC1[Title/Abstract]) OR ASIC2a[Title/Abstract]) OR ASIC2b[Title/Abstract]) OR ASIC2[Title/Abstract]) OR ASIC3[Title/Abstract]) OR ASIC4[Title/Abstract])

The search terms were modified according to the specific vocabulary map of each database.

### Inclusion/exclusion criteria

The selection criteria for studies to be considered for reviewing were as follows:

- Articles were published in English;
- Articles were with main purpose of descriptions of the significance of ASICs in IVDD;
- Articles were about basic science research;

Studies were excluded if:

- Article types were inappropriate, such as review, meta-analysis, and so on;
- The research objects were not intervertebral disc;

### Study selection

Two reviewers independently screened the studies by reviewing the titles and abstracts that met the eligibility criteria and then selected researches according to our inclusion and exclusion criteria. Conflicts in opinions between investigators were resolved by consensus and consultation with the first authors. The references of all publications were also retrieved to obtain possible studies.

### Information extraction

Data relating to study characteristics were collected, and integrated in a conclusive chart to overview the basic feature of this review. Basically, two reviewers independently extracted the description of correlation between ASICs and IVDD from the included studies with a preliminary sorting. Any diagreements were resolved by discussion and consensus.

## Results

### Study selection and characteristics

**Figure 1.**
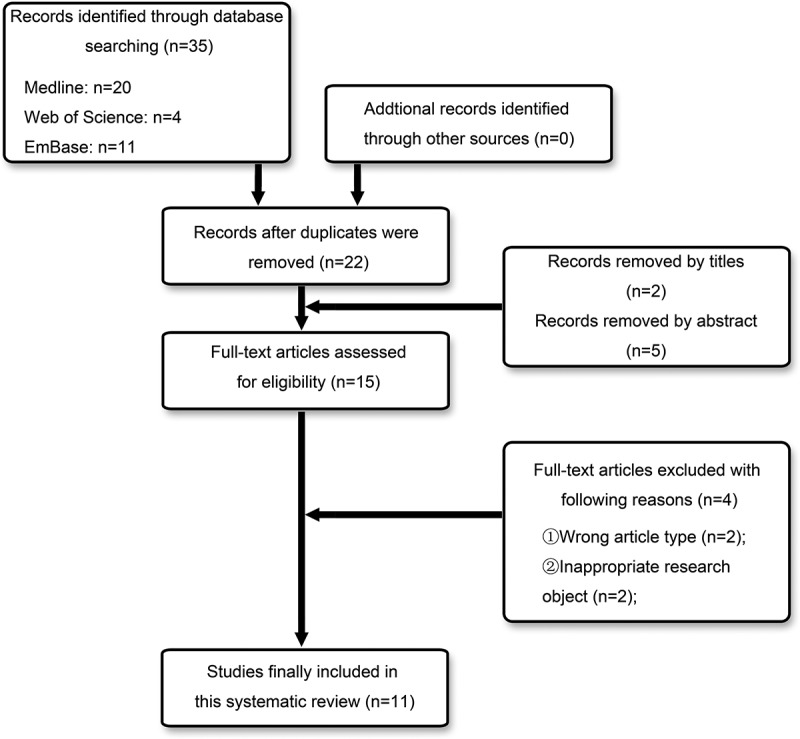
Flowchart for identification and inclusion of relevant studies.

The initial database search identified a total of 35 records, and the removing of duplicates left us with 22 studies preliminarily. After the titles and abstracts were reviewed, 7 of them were eliminated. A full-text review was evaluated in the 15 records maintained, and 4 of them were excluded because of they didn’t meet the inclusion criteria. Finally, 11 articles, meeting the inclusion criteria, were included in the present systematic review. Figure 1 shows the selection process, and the information and characteristics of the included studies are listed in Table 1.

**Table 1.** Characteristics of included studies.

References	Authors	Researching Points		Journal
		ASICs	IVD	
11	Yoshiyasu et al. 2007	ASIC3	NP	Journal of Bone and Mineral Research
12	Yoshiyasu et al. 2008	ASIC3	NP	Journal of Bone and Mineral Research
13	Antonio et al. 2014	ASIC1a, ASIC1b, ASIC2a, ASIC2b, ASIC3, ASIC4	AF, NP	Connective Tissue Research
14	Li et al. 2014	ASIC1a	EP	Expert Opinion on Therapeutic Targets
15	Sun et al. 2014	ASIC1a, ASIC1b, ASIC3	NP	Acta Biochimica et Biophysica Sinica
16	Antonio et al. 2015	ASIC2a, ASIC2b	AF, NP	International Journal of Clinical and Experimental Pathology
17	Cai et al. 2016	ASIC1a	NP	Iranian Journal of Basic Medical Science
18	Gilbert et al. 2016	ASIC3	NP	Scientific Report
19	Yuan et al. 2016	ASIC1a	EP	Cell Stress and Chaperones
20	Xie et al. 2018	ASIC1a	NP	BioResearch Open Access
8	Wang et al. 2019	ASIC3	NP	Biomedicine & Pharmacotherapy

***NOTE.*** ASIC, acid-sensing ion channel; IVD, intervertebral disc; NP, nucleus pulposus; EP, endplate; AF, annulus fibrosus.

### The expression of ASICs in intervertebral disc

Significance of this ion channel family depends greatly on its distribution pattern and expression range in various tissues in vivo [6].

Yoshiyasu firstly reported, in rats, there was the expression of ASIC3 in the AF and NP, both mostly localized to the plasma membrane [11,12]. Furthremore, its expression in NPC was maintained by nerve growth factor (NGF) through p75NTR and extracellular signal-regulated kinase (ERK) signaling pathway [11], while transforming growth factor (TGF)-β, the classical degeneration inducer of NPC, inhibited its expression via SMAD3, a transcriptional repressor [12].

Li’s research focused on the EP and found that the ASIC1a expression was abundant [13].

Recently, Antonio et al. reported [14] that, in healthy IVD, there were detectable levels of ASICs mRNAs that increased along with degeneration, among which the highest variation in expression was observed with ASIC2. Furthermore, in IVDD, there were also increased percentage of ASIC1 and AISC4 positive cells in AF and increased density of cells expressing ASIC1, ASIC2 and ASIC3 in the NP tissue [9,14,15]. The general expression characteristics of ASICs in IVD were listed in Table 2.

**Table 2.** Expression changes of ASICs in intervertebral disc.

ASICs isotype	NP	AF	EP	Relative references
ASIC1a	↑	↑	→	[13,14,16,17,19]
ASIC1b	↑	↑	Unreported	[13, 15]
ASIC2a	↑	→	Unreported	[14,15]
ASIC2b	↑	→	Unreported	[14,15]
ASIC3	↑or↓	→	Unreported	[12,14,19]
ASIC4	↑	↑	Unreported	[14]

*NOTE.* ASIC, acid-sensing ion channel; NP, nucleus pulposus; AF, annulus fibrosus; EP, endplate; ↑, upregulation; ↓,downregulation; →, unchanged;

### NP degeneration

In Yoshiyasu’s in 2007 [11] study exploring the functional significance of ASICs, after the inhibition with Amiloride, they found a profound decrease in NPC viability in both hypertonic medium and acidic culture conditions, which were similar to the NP tissue microenvironment. Moreover, they proved that the death of NPC was mediated by apoptosis and the inhibition of ASICs’ function would promote apoptosis [11].

Among the ASIC isotypes, ASIC1a had been attracted much attention because of its ability to mediate the Ca^2+^ influx ([Ca^2+^]i) and endoplasmic reticulum (ER) stress, which would activate the apoptosis process. With Psalmotoxin (PcTx1), the specific inhibitor of ASIC1a, the increase of [Ca^2+^]i and necrosis in NPC induced by acid were blocked [16,17] (Table 3).

**Table 3.** Functional description of ASICs in intervertebral disc degeneration.

ASIC Isotypes	Functional Description	References
ASIC1a	ASIC1a activation in endplate chondrocytes may trigger Ca^2+^-dependent protease activity and signaling, which leads to apoptosis of endplate chondrocytes in IVDs.	[13]
	ASIC1a activation induced the increased [Ca[^2]^^+^]i of NPCs, which resulted in cell apoptosis and stress-induced premature senesence.	[16]
	ASIC1a is involved in matrix metabolism of endplate chondrocytes under extracellular acidic conditions via NF-kB transcriptional activity.	[20]
	ASIC1a partly regulates ER stress and promotes apoptosis of NPCs under acid stimulus.	[17]
ASIC3	ASIC3 is needed for adaption of the NPCs and annulus fibrosus cells to the acidic and hyperosmotic microenvironment of the IVD.	[11]
	ASIC3 was involved in adapting disc cells to their hydrodynamically stressed microenvironment, suggesting an additional role for this ASIC subunit.	[19]
	Acidic pH causes a catabolic and degenerated phenotype in NPCs which is inhibited by blocking ASIC3 activity, suggesting that this may be a useful therapeutic target for treatment of IVD degeneration.	[18]
	ASIC3 over-expression inhibited the proliferation, arrested cell cycle in G1 phase, promoted the apoptosis in NPCs.	[8]

***NOTE.*** ASIC, acid-sensing ion channel; IVD, intervertebral disc; NPC, nucleus pulposus cell.

Yoshiyasu [11] hypothesized that ASIC3 is needed for adaptation of the NPC to the acidic and hyperosmotic microenvironment, unfortunately, without scientific authentication. On the contrary, Hamish proved that inhibition of ASIC3 would prevent the acid-induced proinflammatory and pain-related/degenerative phenotype in NPC, suggesting that ASIC3 may be a useful therapeutic target for the treatment of IVDD [18]. On the whole, ASIC1a may regulate the survival of NPC, while ASIC3 would accommodate the phenotype (Table 3).

Non-steroid anti-inflammatory drugs (NSAIDs) were well-known non-specific inhibitors of ASICs, while Sun found that the NSAIDs could also regulate the expression of them in NPC [19]. After Ibuprofen treatment, the expression and current of ASIC1 and ASIC3 induced by acidic stress were suppressed, and the degeneration-induced cell death could also be prevented [19] (Table 3).

### EP degeneration

EP tissue belongs to the hyaline cartilage, and as proved in articular cartilage that the hypoxia would upregulate the ASIC1a and leads to the injury of chondrocytes [6], Li and his colleagues [13] tried to confirm the similar function of ASICs in EP. By mediating the [Ca^2+^]i elevation, the ASIC1a’s activation triggers the apoptosis process, and after the specific inhibition of ASIC1a with PcTx-1 or ASIC1a-siRNA, the acidic-induced apoptosis of EP chondrocytes was significantly suppressed [13]. Furthermore, Yuan [20] newly reported that ASIC1a was involved in acid-induced inhibition of Aggrecan (ACAN) and type II collagen (Col2a1) synthesis in EP chondrocytes and acid-induced increase in matrix metalloproteinase (MMP) expression via the NF-kB pathway (Table 3).

### AF degeneration

As listed before, the expressions of ASIC1 and ASIC4 were significantly upregulated in AF of IVDD [14,15], reminding us, with little functional information of ASIC4, that degeneration of AF would be associated with ASIC1. Furthermore, Yoshiyasu [11] once suggested that ASIC3 would adapt the AF cells to the acidic and hyperosmotic microenvironment. Unfortunately, there is little information available about the AF degeneration and ASICs, which may be due to the special collagenous structure of the AF (Table 3).

## Discussion

The unique microenvironment of IVD, with the ischemia and hypoxia, is responsible for its vulnerability and degeneration, but how does the microenvironment correlates with IVDD? Here we noticed the ASICs, which could be responding to the changes in environmental pH and mediate the acid-sensitive ion currents. Only a few researching groups are focusing on the ASICs in IVDD worldwide, but positive results reminded us that the association between ASICs and IVDD could be clinically significant. Actually, the significance has been, to some degree, proved in the arthritis-related research with that articular cartilage is also vulnerable under pathological hypoxia [21]. Physiologically, articular chondrocytes are submerged in the ischemic environment, and the inflammation would aggravate the local acidosis [22], which in turn leads to the activation of ASIC1a and chondrocytes injury [6].

**Figure 2.**
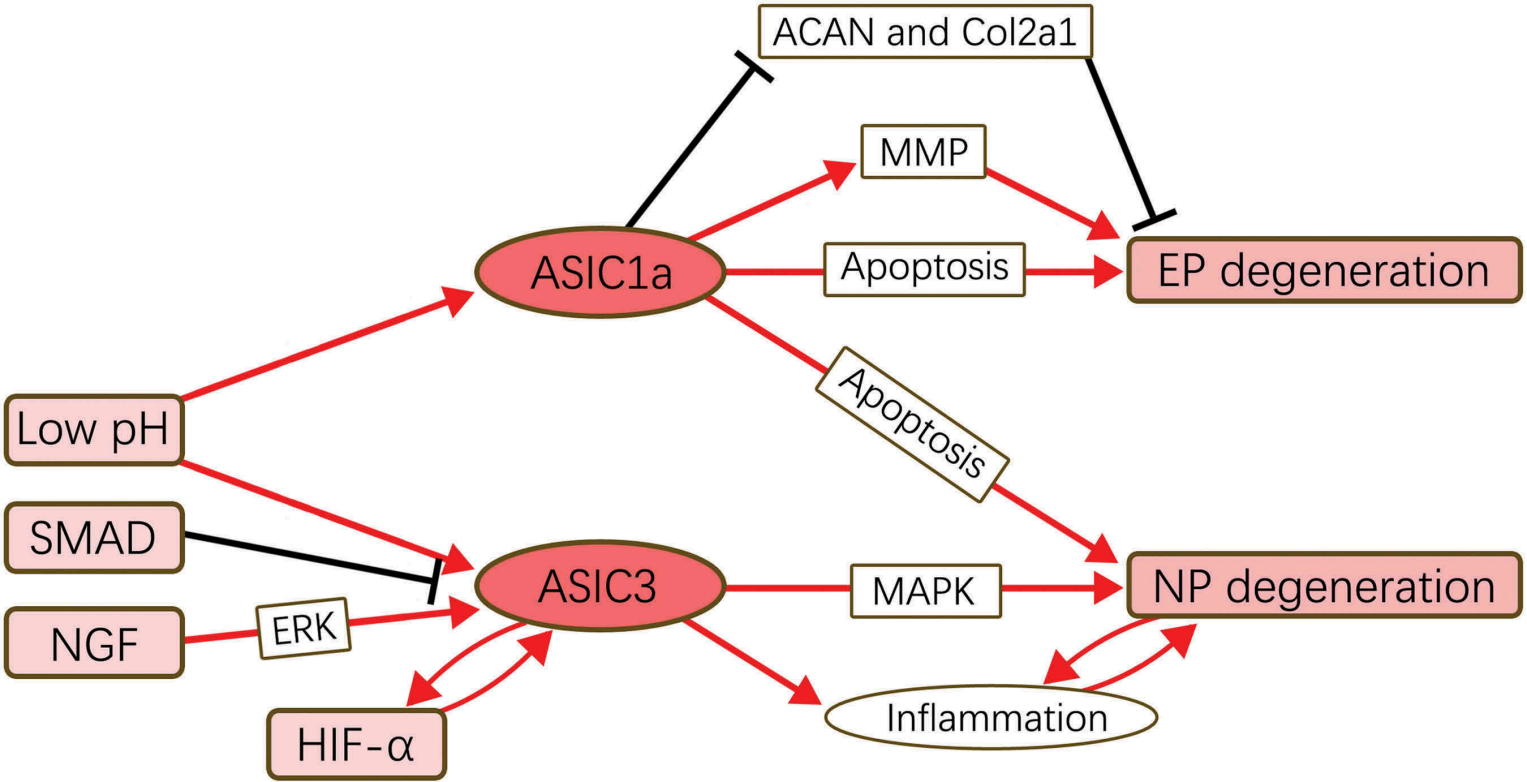
Functional pathways of ASICs in IVDD. ASIC1a mediates the degeneration of EP and NP by regulating the cellular metabolism and apoptosis process. ASIC3 promote the degeneration only in NP possibly via MAPK pathway and its pro-inflammatory function. Low pH is an acceptable up-stream promoter for ASICs’ expression, and SMAD, NGF, and HIF-1α are reported to be the regulator of ASIC3 in IVDD.

In this systematic review, we found that, along with the degeneration, the expression levels of ASICs vary more or less (Table 2). Because of the in-depth study in other field, such as glioma, much attention here has been put into the ASIC1a and ASIC3, and actually, these two isotypes indeed play great roles in the IVDD mechanism. As listed in Figure 2, we concluded of possible regulation mechanism network of ASIC1a and ASIC3 in IVDD with the available results. ASIC1a functions as an apoptosis mediator for cells in EP and NP, with whose activation the apoptosis process would be accelerated. Moreover, ASIC1a’s activation could also regulate the expression of ACAN, Col2a1, and MMP in chondrocytes of EP, facilitating the degeneration. ASIC3, as reported, would only promote the degeneration in NP, possibly via its pro-inflammatory effect. Several factors, including low pH, SMAD, NGF, and hypoxia-induced factor 1a (HIF-1a), would regulate the expression of ASICs upstream, while little information could be extracted here, reminding us the research in upstream regulation of ASICs in IVDD would have research value.

The distribution of ASICs in nucleus pulposus, endplate, annulus fibrosus, and the particular functions of ASIC1a and ASIC3 remind us about the pathological significance of ASICs in IVDD. However, it is important to note that IVDD is not the disease caused by a single etiological factor, but a combination of multiple factors. The elucidation of ASICs-related mechanism could be a small but necessary step forward, which increases the possibility of the complete prevention or cure for IVDD.

## Limitation

Several limitations exist in the current study. Firstly, with only a few studies talking about the function mechanism of ASICs, we can only get a relative simple conclusion in this part. Secondly, the roles played by ASIC2a and ASIC2b were barely mentioned.
